# Neglected Sprengel’s deformity in an 80-year-old female cadaver: a case report

**DOI:** 10.1186/s13256-024-04528-w

**Published:** 2024-04-28

**Authors:** Shun Otsuka, Yuki Tamaki, Masaki Matsushita, Naoyuki Hatayama, Munekazu Naito

**Affiliations:** 1 Department of Anatomy, Aichi Medical University, 1-1 Yazakokarimata, Nagakute, Aichi 480-1195 Japan; 2 Department of Orthopaedic Surgery, Nagoya University Graduate School of Medicine, 65 Tsurumai-cho, Showa-ku, Nagoya, Aichi 466-8550 Japan

**Keywords:** Sprengel’s deformity, Cadaveric study, Klippel–Feil syndrome, Omovertebral bone, Case report

## Abstract

**Background:**

Sprengel’s deformity is a congenital abnormality of the shoulder girdle. Because scapular retraction, such as the Green procedure, is usually performed during childhood to improve esthetics and shoulder function, Sprengel’s deformity is rarely found in older patients.

**Case presentation:**

We presented a unique case of a Japanese female cadaver with Sprengel’s deformity at the age of 80 years. Anatomical dissection and radiological imaging revealed musculoskeletal anomalies associated with Sprengel’s deformity, including Klippel–Feil syndrome, presence of an omovertebral bone, and absence of the trapezius muscle. In addition, bilateral cervical ribs were in contact with the brachial plexus. These anomalies may lead to numbness, pain, and limited range of motion of the neck and upper girdle with aging.

**Conclusions:**

Because most adult patients with Sprengel’s deformity experience neck pain and limited movement of the shoulder, the presented case is a rare case of neglected Sprengel’s deformity in an 80-year-old cadaver.

## Background

Sprengel’s deformity is a common congenital abnormality of the shoulder girdle caused by an undescended scapula from the neck to the posterior thorax during embryonic development [1, 2]. The pathogenesis of Sprengel’s deformity is related to a primary genetic abnormality in the organization of the mesenchymal precursor of the cervical spine between the third and seventh gestational weeks [3]. Sprengel’s deformity is usually associated with Klippel–Feil syndrome and muscle defects around the shoulder girdle, which are caused by pathologic lesions early in embryonic gestation [4, 5]. The omovertebral bone, which is an abnormal connection between the posterior cervical spine and the superomedial border of the scapula, is often found in patients with Sprengel’s deformity. Several possible hypotheses have been put forward for the etiology of the existence of the omovertebral bone, including overgrowth of the spinous process or scapula in the fetal period and ossification of the connective tissue in the intermuscular planes between the spine and scapula [3, 5]. Because the shoulder in patients with Sprengel’s deformity cannot be elevated during passive movements and the omovertebral bone can cause local pain, scapular retraction with the extraction of the omovertebral bone is usually performed around the age of 6 years to improve esthetics and shoulder function [6]. Although some cases of Sprengel’s deformities have been reported in adult patients, no data have been available in older cadavers. Herein, we report a unique case of an 80-year-old female cadaver with Sprengel’s deformity and associated abnormalities.

## Case presentation

The cadaver was a Japanese female donated to Aichi Medical University for teaching and research purposes. The cause of death was senility at the age of 80 years, and the patient had a history of lower gastrointestinal bleeding and dementia. Anatomical dissection and observation were performed for medical students to see the presence or location of the bones, muscles, and nerves.

**Fig. 1:**
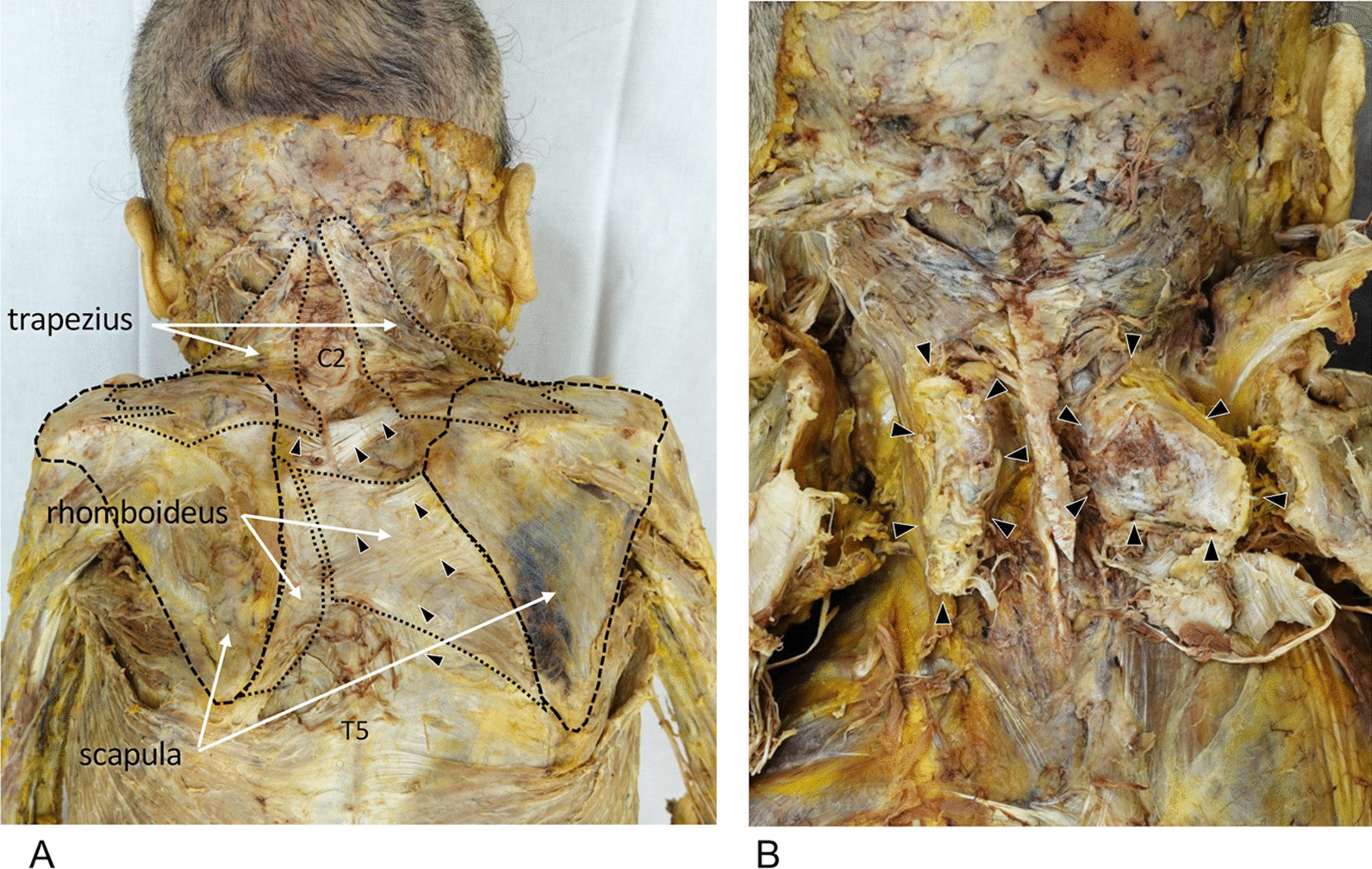
Posterior view of the neck and shoulder after removal of the skin.** A** Posterior view of the neck and shoulder after removal of the skin and subcutaneous adipose tissue. Bilateral scapulae were located between C2 and T5 spinous processes with the absence of the ascending part of the trapezius and hypoplasia of the left rhomboideus. Tight fascial tissues covered the muscles (arrowhead). **B** Posterior view of the neck after removal of the fascia, trapezius, and rhomboideus. Omovertebral bones were observed bilaterally (arrowhead)

Both scapulae were highly elevated at the level between the second cervical (C2) and the fifth thoracic (T5) spinal processes (Fig. 1A). The trapezius was bilaterally absent in the ascending portion, and the left rhomboideus muscle was hypoplastic. Many fascial fibers ran from the medial border of the scapula to the spinous process on the muscles. The omovertebral bone was present bilaterally, and the surface was covered with tight fascial tissue (Fig. 1B). An articular disk was found between the right omovertebral bone and the scapula. Based on the higher position of the scapulae with omovertebral bones, this cadaver was diagnosed with Sprengel’s deformity.

**Fig. 2:**
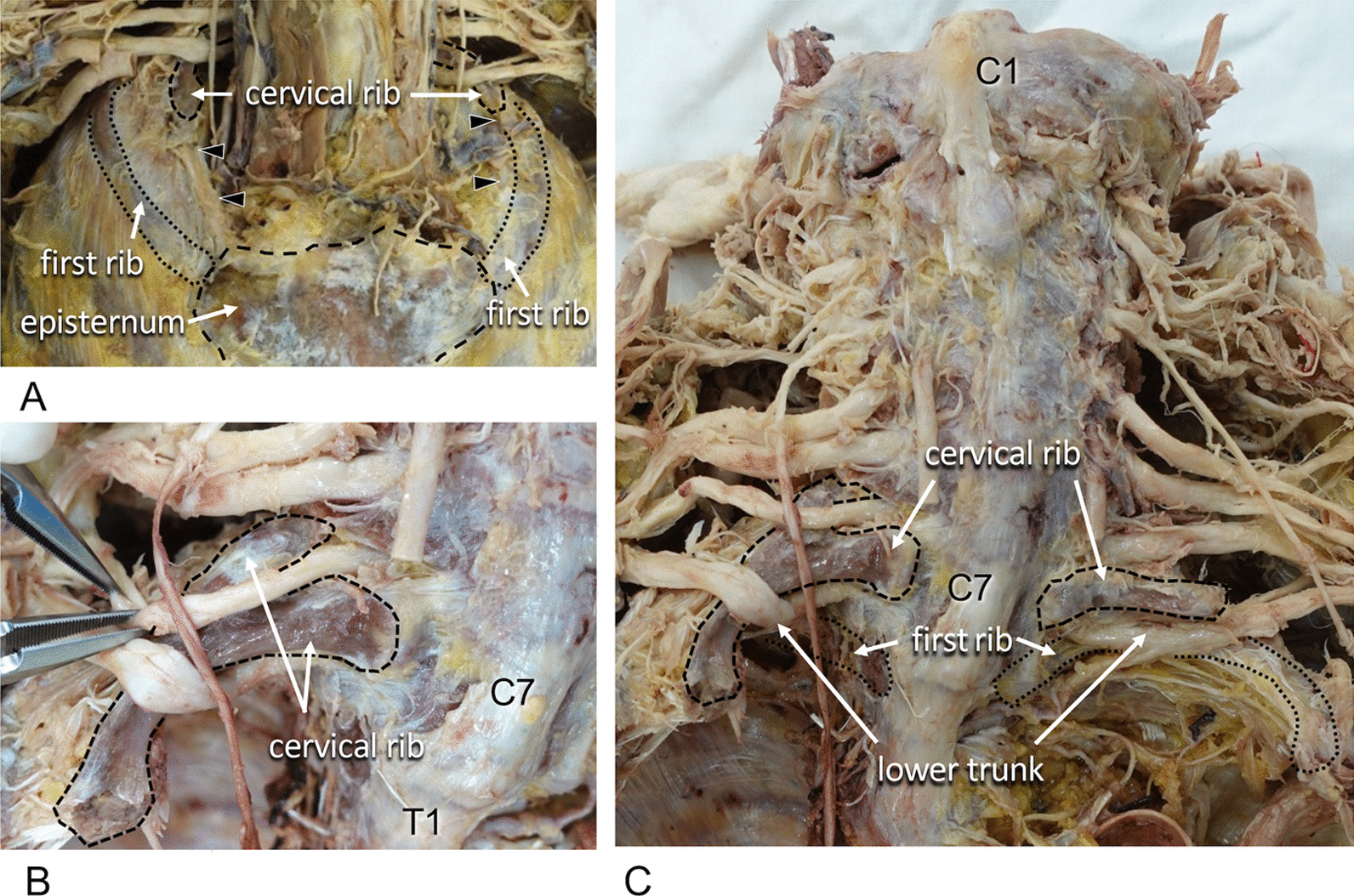
Relationships between the cervical rib and the cervical nerve.** A** Anterior view of the cervical and thoracic regions. Cervical ribs were connected to the episternum by ligamentous fibers (arrowhead). **B** Anterior view of the cervical region. Tweezers indicated the seventh cervical nerve. The right bisected cervical rib was enclosing the cervical nerve root. **C** Anterior view of the whole cervical region. The lower trunk of the brachial plexus was interposed between the cervical rib and the first rib

Bilateral cervical ribs attached to the episternum via ligamentous fibers were observed (Fig. 2A). The proximal site of the right cervical rib, which was bisected in contact with C6 and C7, enclosed the seventh cervical nerve root (Fig. 2B). Cervical ribs were located between the middle and lower nerve trunks of the brachial plexus (Fig. 2C). There were 11 thoracic vertebrae and 11 pairs of ribs. No abnormality was observed in the innervation of the neck and upper extremity muscles.

**Fig. 3:**
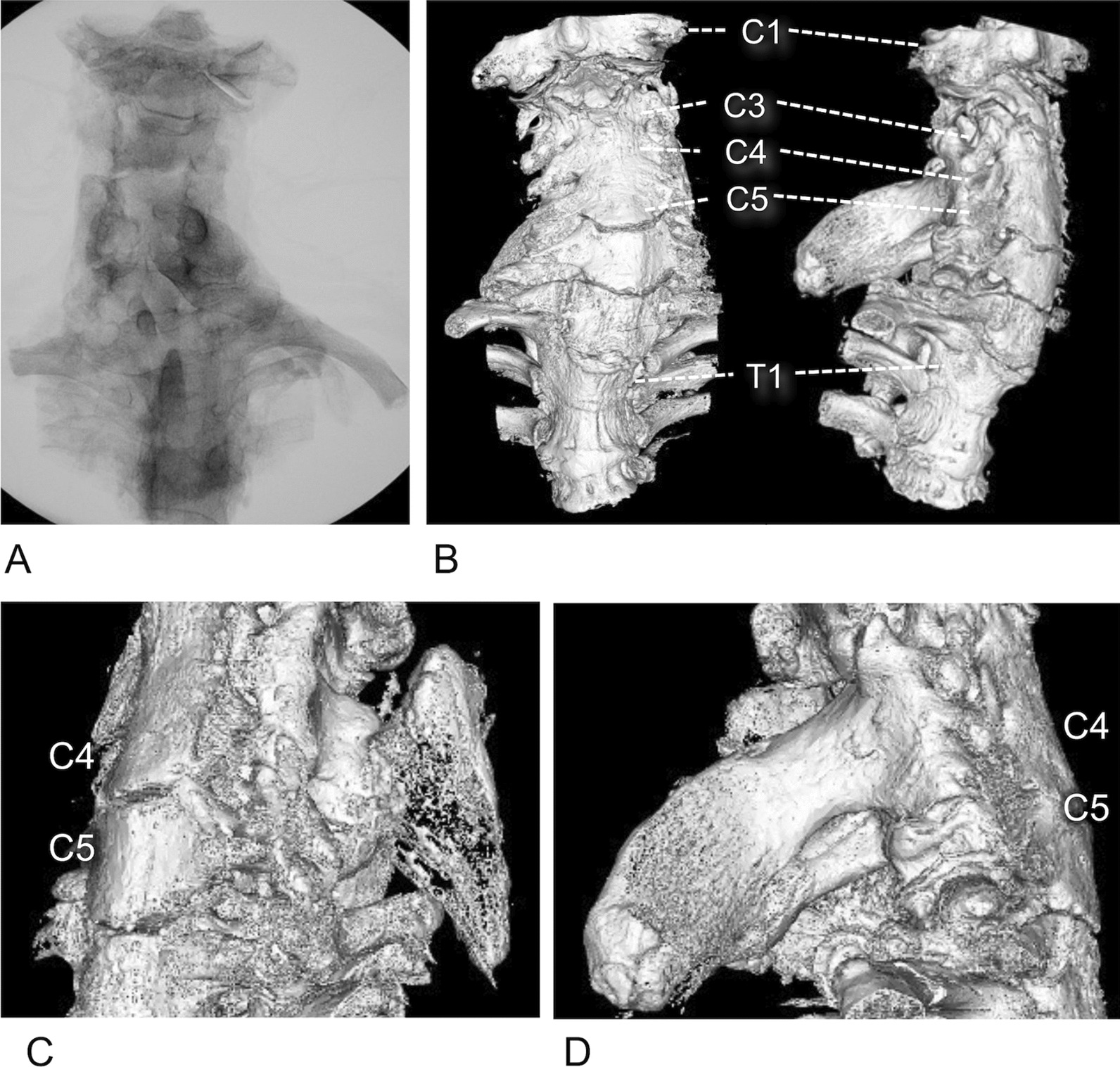
X-ray and computed tomography (CT) images of cervical and thoracic regions.** A** Posteroanterior X-ray image of the vertebrae (C1–T2). **B** Anterior and lateral views of the three-dimensional (3D) reconstructed CT scan of the vertebrae (C1–T2). Many vertebrae are deformed and fused. **C** and** D** 3D reconstructed CT images of the omovertebral bone on the left (C) and right sides (**D**)

Radiograph showed cervical scoliosis with deformed vertebral body (Fig. 3A). A subsequent computed tomography scan with three-dimensional reconstruction was used to identify the associated bone abnormalities in the cervical and upper thoracic regions (Fig. 3B). Spinous processes were fused in C4–C5, C6–C7, and T1–T2. The vertebral bodies were also fused between C3 and C5. The omovertebral bones were located on the spinous processes of C4 and C5 on the left side (Fig. 3B, C) and were inseparably fused to C3, C4, and C5 on the right side (Fig. 3B, D). The cadaver was confirmed to have Sprengel’s deformity associated with Klippel–Feil syndrome [7].

## Discussion and conclusion

This is a rare case of a cadaver with bilateral Sprengel’s deformities at the age of 80 years. Most cases of Sprengel’s deformity are associated with Klippel–Feil syndrome (16–27%), scoliosis (35–55%), rib anomalies (16–48%), and omovertebral bone (20–50%) [5, 8, 9]. Because these abnormalities were observed, our cadaver was diagnosed with Sprengel’s deformity despite the advanced age.

Because a part of the trapezius defect has been rarely reported in cadavers without any anomalies [10], the loss of the ascending portion of the trapezius led to the diagnosis of Sprengel’s deformity in the present case. Absent or hypoplastic muscles around the shoulder, including the trapezius, pectoral, sternocleidomastoid, and serratus anterior muscles, have been reported in patients with Sprengel’s deformity [8, 11, 12]. The ascending portion of the trapezius muscle mainly contributes to the scapular descent. The loss of the trapezius muscle may aggravate the elevation of the scapula and limited range of motion due to Sprengel’s deformity.

In older patients, scapular retraction, such as the Green and Woodward procedures, poses a risk of compression or entrapment of the brachial plexus [13]. In the present case, the brachial plexus was interposed between the cervical ribs and the first ribs. Early surgical treatment is desirable for patients with Sprengel’s deformity because the entrapment of the brachial plexus may cause arm numbness and movement limitations. Several case reports of adult patients with Sprengel’s deformity have described only the resection of the omovertebral bone and upper scapula spine, although scapular retraction could be also performed in younger patients. These surgical results for adult patients have not been consistent in improving pain, range of motion in the shoulder joint, and cosmetic deformity (Table 1) [14–19]. Because most adult patients with Sprengel’s deformity may experience neck pain and limited movements of the shoulder, Sprengel’s deformity in an 80-year-old cadaver is considered a neglected case.

**Table 1:** Previous case reports of an adult patient with Sprengel’s deformity

Age	Sex	Side	Surgical operation	Results	References
20	Female	Left	Resection of the left omovertebral bone	Reduced neck pain, slightly improved shoulder range of motion, and improved cosmetic deformity	[14]
26	Female	Bilateral	Resection of the left omovertebral bone	Reduced neck pain and improved shoulder range of motion and cosmetic deformity	[14]
22	Female	Left	Resection of the left omovertebral bone and superior angle of the scapula	Reduced neck pain, improved shoulder range of motion, and unchanged asymmetry of the shoulder girdle	[15]
34	Female	Left	Resection of the left omovertebral bone and decompression of the spinal canal	Reduced neck pain, improved neck range of motion and cosmetic deformity, and unchanged neurological findings	[16]
50	Female	Left	Resection of the left omovertebral bone and decompression of the spinal canal	Reduced neck pain, improved neck range of motion and cosmetic deformity, and unchanged neurological findings	[17]
25	Female	Left	Resection of the left omovertebral bone	Reduced neck pain and improved shoulder range of motion	[18]
19	Female	Bilateral	Resection of bilateral omovertebral bones and detachment of the trapezius and rhomboid muscle origins	Improved range of motion in the shoulder and disabilities of the arm, shoulder, and hand	[19]

The information presented is limited because observations were made using a cadaver. First, we did not examine the range of motion of the joints and only performed anatomical dissection and observation. Second, observations were confined to a certain region of the body for educational use and cremation was required because the current case was found by chance during an anatomical dissection course for medical students. Third, no information on neurological symptoms during the lifetime was available, although cervical myelopathy was reported in a middle-aged patient with untreated Sprengel’s deformity [17]. The present cadaver might have neck pain and paralysis.

## Data Availability

Not applicable.
